# From Myzocytosis to Cytostomal Nutrient Uptake and Transport by Intracellular *Plasmodium* Species

**DOI:** 10.3390/pathogens12081036

**Published:** 2023-08-14

**Authors:** Tobili Y. Sam-Yellowe

**Affiliations:** Department of Biological, Geological and Environmental Sciences, Cleveland State University, Cleveland, OH 44115, USA; t.sam-yellowe@csuohio.edu

*Plasmodium falciparum* causes severe and lethal malaria. Approximately 247 million malaria cases occur worldwide, resulting in 600,000 deaths annually, with most deaths in children under five years of age and in pregnant women [1]. Mechanisms of sporozoite and merozoite invasion into hepatocytes and erythrocytes, respectively, and development within the host cell have been the subject of extensive research. In the blood stage, the mechanisms of nutrient uptake across the host red cell membrane, parasitophorous vacuole membrane (PVM) and parasite plasma membrane (PPM) have received much focus due to the trafficking of multiple parasite proteins to the host cell membrane through the Maurer’s clefts, tubovesicular membranes and other mobile membranous vesicles like the J-dots [2,3]. The transport of nutrients from the extracellular environment into the host erythrocyte and from the host cell cytoplasm into the parasite, although significant, have received less attention, in part, due to the challenges of investigating the trafficking pathways. *Plasmodium falciparum* modifies the erythrocyte membrane by laying down transport channels such as the plasmodial surface anion channel (PSAC) used for metabolite and ion transport. Upon merozoite infection of the host erythrocyte, the erythrocyte membrane becomes increasingly permeable [4] as various nutrients from the external environment of the host cell are transported into the growing parasite [4]. Patch–clamp studies have confirmed the presence of the channel in the infected erythrocyte membrane. However, factors contributing to solute selectivity and uptake across PSAC are unknown [4]. 

CLAG rhoptry proteins associate with the high-molecular-weight rhoptry proteins RhopH2 and RhopH3 to form the high-molecular-weight complex RhopH, which inserts itself into the erythrocyte membrane following merozoite invasion. The complex participates in PSAC activity in all *Plasmodium* species studied [3,4]. However, how the channel forms and the specific contributions of each RhopH protein to the function of the channel remain elusive, and are an area that warrants continued investigation.

The uptake of hemoglobin from the host erythrocyte cytoplasm occurs, with up to 80% of the host cell cytoplasm taken up by intracellular *Plasmodium* species [5]. The contents of the host cell cytosol are transported across the parasite membrane, and digested to release amino acids from hemoglobin with the formation of hemozoin pigment. Since not all amino acids are obtained from hemoglobin, essential amino acids such as isoleucine are obtained extracellularly [5]. Among the Apicomplexa, host cell cytosol uptake (HCCU) is required for parasite growth and pathogenicity [6]. In *Plasmodium* species, the transport of nutrients into the parasite is thought to take place at the PVM/PPM interface and proceeds through the endocytic pathway into vacuolar compartments, followed by digestion using proteases [6]. In addition to hemoglobin, essential amino acids, glucose, vitamins, purine precursors and some lipids are taken up by the intracellular parasite [6,7]. During the growing blood stage, the parasite modifies the host erythrocyte extensively to accommodate trafficking pathways. While there is a growing body of evidence from microscopy and the recent identification of markers showing the involvement of endosomal trafficking, the mechanisms of nutrient uptake and trafficking remain elusive, in particular at the PVM/PPM membrane interface where the cytostome has been described as the structure used for endocytosis [5,6,7,8]. 

The cytosotome is an invagination of the PVM/PPM, and hemoglobin containing vesicles originating from the cytostome have been observed to be trafficked to the digestive vacuole [5,6,7,8]. Aikawa et al. [9], in ultrastructural studies, described and coined the name “cytostome” (also known as micropyle and pellicular pore) for a structure they identified in the pellicular membrane that was active in host cell cytoplasm uptake, termed “intracellular phagotropy” at the time [9]. The food vacuole was described to enlarge from the cytostome [9]. Ring-stage parasites have been reported to form smaller food vacuoles that coalesce to form the large digestive vacuole seen in the trophozoite stages [7,10]. The identification of VPS45 in *P. falciparum*, as a protein involved in the trafficking pathway, in particular, and in membrane fusion, paves the way for additional proteins to be identified with roles in cytostome function. VPS45 is the first protein shown to be directly involved in HCCU [10]. In experiments where VPS45 was inactivated in knock-sideways parasites, host cell cytoplasm delivery to the food vacuole was blocked and phosphatidylinositol 3-phosphate (PI(3)P) was identified on food vacuole membranes and HCCU vesicles [10]. The uptake of the host cytoplasm was confirmed in host cells pre-loaded with fluorescent dextran, where the dextran was identified in vesicles inside the intracellular parasite [10]. The continued use of conditional mutants, fluorescent reporters and other membrane tags promise to reveal the mechanisms underlying nutrient uptake and the proteins involved.

Artemisinin resistance is associated with endocytosis at the cytostome [5,6,7,8]. Proteins identified as having roles in hemoglobin uptake across the PVM/PPM interface include epidermal growth factor receptor substrate-15 (Eps 15), ubiquitin carboxyl-terminal hydrolase (UBP1), adaptor protein-2µ (AP-2 µ) and Kelch-13. Gene disruptions of the genes encoding these four proteins decreases hemoglobin uptake [5,6,7,8]. Both AP-2 µ and Eps-15 have roles in endocytosis but their function in *Plasmodium* species is unclear. Proteins identified as having a role in endocytosis in *Toxoplasma gondii* and *P. falciparum* include non-canonical proteins that function only among apicomplexans. Endocytosis and other mechanisms of nutrient uptake across the PPM are poorly understood and the proteins involved are largely unknown. 

Kelch 13 is present in all apicomplexans and myzozoans and is associated with endocytosis [11]. The conserved coiled-coil sequence of Kelch 13 represents an ancient conserved structure associated with endocytosis [11]. Whether endocytosis is only used for nutrient uptake or for other functions such as membrane homeostasis is unclear and needs further investigation in apicomplexans and myzozoans. The inner membrane complex (IMC) is found in all alveolates, which includes the apicomplexans and colpodellids [11]. In *Plasmodium*, the IMC is disassembled to enable endocytosis across the PPM and during schizogony, and in merozoites, the IMC is reassembled [11,12]. Nutrient uptake occurs through myzocytosis among the free-living close relatives of Apicomplexans, such as the predatory *Colpodella* species [12], although these processes are poorly understood. The processes of myzocytosis and endocytosis among *Colpodella* species may occur in an area of the IMC also disassembled for nutrient uptake. The “myzocytotic aperture” present in the rostrum within the pseudoconoid of *Colpodella* sp. is used for the uptake of the prey’s plasma membrane into the cytoplasm of *Colpodella* sp. before the degradation of the membrane. This aperture may represent a structure similar to the cytostome [13]. Once inside the predator’s cytoplasm, the prey’s cytoplasmic contents are aspirated across a tubular tether into the posterior food vacuole of the predator [13]. The resemblance of the myzocytic aperture to the cytostome remains speculative and will require experimental studies for verification. 

This introductory Editorial highlights areas where much progress has been made to understand nutrient uptake in *Plasmodium* species. However, many important questions remain regarding the fundamental processes involving the nature of the cytostome, its role in nutrient uptake, the proteins involved and food vacuole formation. The transport of nutrients into the food vacuole (digestive vacuole, DV) occurs through a process similar to endocytosis across the membrane of the DV, which has been shown to contain different transporters and channels. Transporters such as multidrug resistance proteins 1 and 2 (MDR1, 2), amino acid transporter (AAT), chloroquine resistance transporter (CRT) and vacuolar-type adenosine triphosphatase (V-ATPase) have been identified on the digestive vacuole membrane. However, many of the proteins associated with transporter activity across the digestive vacuole membrane are unknown [5] and the DV remains an important target of antimalarial drugs. 

Finally, for this Special Issue, “Nutrient Uptake and Trafficking in *Plasmodium* Species”, various articles addressing nutrient uptake across the host erythrocyte membrane, hepatocyte membrane, PVM, PPM and DVM in the blood and liver stage are expected. These articles will provide a clearer understanding of these transport processes, which are fundamental to the biology of *Plasmodium* species, and will also stimulate new avenues for research. Some of the same molecules and structures participating in nutrient transport in the blood stage are found in the liver stage. For example, exported protein 1 and 2 (EXP1 and EXP2) in the PVM and the tubovesicular network function in the liver stage. Robust lipid and metabolite uptake occurs in the liver stage due to the thousands of merozoites produced during schizogony [14]. The types of molecules and markers functioning at the host–parasite interface for the uptake of cholesterol, lipoic acid, phosphatidylcholine, amino acids and glucose, and the mechanisms of molecular transport remain unclear in the hepatocyte and parasite [14]. Trafficking pathways present important targets for antimalarial drugs. Studies in myzozoans including the chrompodellids are also expected for this Special Issue, as these phylogenetically close relatives of the apicomplexans might hold significant clues to the evolutionary origins of the nutrient uptake mechanisms in *Plasmodium* species.

## References

[1] World Health Organization WHO (2022). World Malaria Report 2022.

[2] El Chamy Maluf S., Icimoto M.Y., Melo P.M.S., Budu A., Coimbra R., Gazarini M.L., Carmona A.K. (2020). Human plasma plasminogen internalization route in Plasmodium falciparum-infected erythrocytes. Malar. J..

[3] Desai S.A. (2022). Epigenetics of malaria parasite nutrient uptake, but why?. Trends Parasitol..

[4] Desai S.A. (2014). Why do malaria parasites increase host erythrocyte permeability?. Trends Parasitol..

[5] Matz J.M. (2022). *Plasmodium*’s bottomless pit: Properties and functions of the malaria parasite’s digestive vacuole. Trends Parasitol..

[6] Elsworth B., Keroack C.D., Duraisingh M.T. (2019). Elucidating Host Cell Uptake by Malaria Parasites. Trends Parasitol..

[7] Edgar R.C.S., Counihan N.A., McGowan S., de Koning-Ward T.F. (2022). Methods Used to Investigate the *Plasmodium falciparum* Digestive Vacuole. Front. Cell Infect. Microbiol..

[8] Spielmann T., Gras S., Sabitzki R., Meissner M. (2020). Endocytosis in *Plasmodium* and *Toxoplasma* Parasites. Trends Parasitol..

[9] Aikawa M., Hepler P.K., Huff C.G., Sprinz H. (1966). The feeding mechanism of avian malarial parasites. J. Cell Biol..

[10] Jonscher E., Flemming S., Schmitt M., Sabitzki R., Reichard N., Birnbaum J., Bergmann B., Höhn K., Spielmann T. (2019). PfVPS45 Is Required for Host Cell Cytosol Uptake by Malaria Blood Stage Parasites. Cell Host Microbe.

[11] Koreny L., Mercado-Saavedra B.N., Klinger C.M., Barylyuk K., Butterworth S., Hirst J., Rivera-Cuevas Y., Zaccai N.R., Holzer V.J.C., Klingl A. (2023). Stable endocytic structures navigate the complex pellicle of apicomplexan parasites. Nat. Commun..

[12] Valigurová A., Florent I. (2021). Nutrient Acquisition and Attachment Strategies in Basal Lineages: A Tough Nut to Crack in the Evolutionary Puzzle of Apicomplexa. Microorganisms.

[13] Sam-Yellowe T.Y., Asraf M.M., Peterson J.W., Fujioka H. (2023). Fluorescent Nanoparticle Uptake by Myzocytosis and Endocytosis in *Colpodella* sp. ATCC 50594. Microorganisms.

[14] Nyboer B., Heiss K., Mueller A.K., Ingmundson A. (2018). The *Plasmodium* liver-stage parasitophorous vacuole: A front-line of communication between parasite and host. Int. J. Med. Microbiol..

